# Is gastroesophageal reflux linked to inflammation-related gestational complications and poor obstetric history?

**DOI:** 10.1186/s12884-026-08640-1

**Published:** 2026-01-12

**Authors:** Kemal Beksac, Hanife Guler Donmez, Murat Cagan, Erdem Fadiloglu, Mehmet Sinan Beksac

**Affiliations:** 1 Department of General Surgery, University of Health Science, Ankara Oncology Hospital, Ankara, Türkiye; 2 Department of Biology, Hacettepe University, Faculty of Science, Ankara, Türkiye; 3 Department of Obstetrics and Gynecology, Division of Perinatology, Hacettepe University, Faculty of Medicine, Ankara, Türkiye; 4 Department of Obstetrics and Gynecology, Liv Ankara Hospital, Istinye University, Istanbul, Türkiye

**Keywords:** Gastroesophageal reflux, Poor obstetric history, Pre-conceptional counseling, Inflammation, Autoimmune disorders

## Abstract

**Introduction:**

This study investigated inflammation-related co-morbidities in women with gastroesophageal reflux (GER) and poor gestational outcomes.

**Methods:**

A retrospective cohort of 17 women with GER and 207 without GER who were admitted to a pre-conceptional counseling program was analyzed. All patients were evaluated for the presence of risk factors associated with obstetric complications and poor gestational outcomes, including hereditary thrombophilia, methylenetetrahydrofolate reductase (MTHFR) polymorphisms, type 2 diabetes mellitus, chronic inflammatory diseases, and autoimmune disorders.

**Results:**

GER was present in 7.59% (17/224) of women, and 35.3% (6/17) of GER-positive cases had gastritis and/or chronic peptic ulcer disease. Chronic inflammatory and autoimmune diseases were significantly more frequent in women with GER (*p* = 0.001 and *p* = 0.002, respectively). There was also a statistically significant difference in the distribution of *MTHFR* 677CC, -CT, and -TT genotypes in terms of the presence of GER (*p* = 0.036). A higher frequency of the *MTHFR* 677TT genotype was observed in women with GER.

**Conclusions:**

Presence of GER may be indicative of inflammation-associated “placenta-related obstetric complications” and poor gestational outcomes in subsequent pregnancies.

## Introduction

Gastroesophageal reflux (GER) is a clinical condition characterized by heartburn, acid regurgitation, and reflux of gastric contents into the esophagus [1, 2]. Histopathological findings of reflux-induced esophagitis are important for the diagnosis of GER, and esophagogastroduodenoscopy (EGD) is generally the main diagnostic modality to evaluate this inflammatory process [3, 4].

Beyond a localized esophageal disorder, GER has increasingly been recognized as a condition associated with inflammatory changes in gastroesophageal tissues as well as circulating inflammatory mediators and cytokines. Notably, GER and inflammatory processes appear to be linked in a bidirectional manner, with each condition potentially exacerbating the other [4–8]. GER symptoms and complications may arise through multifactorial mechanisms, and chronic inflammatory diseases and related co-morbidities may play a contributory role in the activation of these etiological factors [9–12].

GER is frequently observed during pregnancy and is often considered a physiological condition related to gestational changes rather than a primary medical disorder. However, if the reflux symptoms are inadequately recognized, then GER may have an adverse effect on the quality of life of pregnant women [13–15]. Therefore, it is important to recognize the presence of GER and to distinguish gestation-induced mechanical reflux from pre-existing GER, which may be associated with inflammation-related co-morbidities known to increase the risk of obstetric complications and perinatal morbidity/mortality [9–12].

It has been demonstrated that chronic inflammatory diseases, autoimmune disorders, and methylenetetrahydrofolate reductase (*MTHFR*) polymorphisms are associated with placenta-related obstetric complications [16–31]. GER is frequently accompanied by inflammatory changes, suggesting a potential overlap with systemic inflammatory conditions. In this context, the identification of chronic inflammatory diseases, autoimmune disorders, and other co-morbidities in patients with GER may be relevant when considering adverse obstetric outcomes. Furthermore, epigenetic and genetic factors related to systemic inflammation may represent possible biological links between GER and poor obstetric history. Previous studies have reported associations between the *MTHFR* 677TT homozygous variant, plasminogen activator inhibitor-1 (PAI-1) alterations, and chronic systemic inflammation [32, 33].

In this study, we evaluated inflammation-related co-morbidities that are known risk factors for obstetric complications in women with GER and a poor obstetric history.

## Materials and methods

This retrospective cohort study included 17 women with gastroesophageal reflux (GER) (study group) and 207 women without GER (control group) who were admitted to a pre-conceptional counseling program for women with “poor obstetric history” between 2016 and 2019 at the Perinatology Outpatient Clinic of Hacettepe University.

Poor obstetric history was defined as the presence of at least one obstetric complication (miscarriage, preeclampsia, fetal growth restriction, preterm birth, and others) and/or poor gestational outcome (APGAR score < 7, neonatal intensive care unit admission, stillbirth, or others) at the previous pregnancy/pregnancies. Data were retrieved from the electronic database of the Division of Perinatology. Ethical approval was obtained before the study (GO19/1064). There were no missing data in the study, and all available variables were included in the statistical analyses.

GER is defined as the presence of esophagitis with heartburn, acid regurgitation, and food reflux (presence of gastric content towards the esophagus) and an inadequate symptom response despite eight weeks of proton-pump inhibitor therapy, which was confirmed by EGD and biopsy. All patients underwent a comprehensive medical evaluation together with a detailed medical anamnesis (including GER). All patients were evaluated in terms of the presence of risk factors such as various systemic disorders, hereditary thrombophilia, plasminogen activator inhibitor-1 (PAI-1), *MTHFR* C677T and A1298C polymorphisms, type-2 diabetes mellitus (DM), chronic inflammatory diseases, and autoimmune disorders, for obstetric complications and poor gestational outcomes.

### Statistical analysis

Statistical analyses were conducted using SPSS v.23 (Statistical Package for the Social Sciences, IBM-SPSS, Chicago, IL, USA). Maternal age, gravida, parity, and number of miscarriages were presented as mean ± standard deviation (SD). The normality of these numerical values was assessed using the Shapiro-Wilk normality test. As only maternal age exhibited a normal distribution, it was consequently compared using an independent t-test. Gravida, parity, and number of miscarriages were compared using the non-parametric Mann-Whitney U test. Categorical data were compared using Fisher’s exact test or Yates’ Continuity test based on expected numbers. A p-value less than 0.05 was considered statistically significant.

## Results

There was no statistically significant difference between the GER-positive and GER-negative groups in terms of demographic and clinical characteristics (*p* > 0.05 for all) (Table 1). As shown in Table 2, 7.59% (17/224) of the study population was GER-positive. Among women with GER, 35.3% (6/17) had concomitant gastritis and/or chronic peptic ulcer disease. Specifically, chronic peptic ulcer was observed in three women (17.6%), gastritis in two women (11.8%), and one woman (5.9%) had both chronic peptic ulcer and gastritis at the time of evaluation.

**Table 1:** The comparison of women with and without GER in terms of maternal age, gravidity, parity, and miscarriage

	GER (+) *n* = 17	GER (˗) *n* = 207	*P*
Maternal age			
* Mean ± SD*	34.5 ± 4.75	33.1 ± 5.07	0.256^a^
Gravidity			
* Mean ± SD*	3.5 ± 1.32	4.2 ± 1.88	0.143^b^
Parity			
* Mean ± SD*	1.8 ± 1.09	1.7 ± 1.01	0.844^b^
Miscarriage			
* Mean ± SD*	1.4 ± 1.37	1.8 ± 1.87	0.340^b^

*GER* Gastroesophageal reflux

a Independent t test

b Mann Whitney U test

**Table 2:** Distribution of gastroesophageal reflux (GER) positivity and GER positivity plus gastritis or/and chronic peptic ulcer among GER positive patients

GER Status	Frequencies (percentages)
*GER (+) only*	11 (64.7)
*GER (+) + Chronic Ulcer*	3 (17.6)
*GER (+) + Gastritis*	2 (11.8)
*GER (+) + Chronic Ulcer + Gastritis*	1 (5.9)
Total (17/224, 7.59%)	17 (100)

*GER* Gastroesophageal Reflux

Table 3 presents a comparison of clinical and biochemical parameters between women with and without GER. The overall co-morbidity rate, defined as the presence of immunological problems (autoimmune disorders and chronic inflammatory diseases), thrombophilia, and type-2 DM, was significantly higher in the GER-positive group (82.4%) compared with the GER-negative group (39.6%) (*p* = 0.002).

**Table 3:** Comparison of clinical and biochemical parameters among women with and without GER

	GER (+) *n* = 17 (%)	GER (˗) *n* = 207 (%)	*P*
*Co-morbidity*	14 (82.4)	82 (39.6)	**0.002** ^b*^
*Immunological problems*	11 (64.7)	23 (11.1)	**<0.001** ^a*^
*Autoimmune diseases*	6 (35.3)	15 (7.2)	**0.002** ^a*^
*Chronic inf. disease*	6 (35.3)	11 (5.3)	**0.001** ^a*^
*DM*	5 (29.4)	28 (13.5)	0.144^**a**^
*Thrombophilia*	2 (11.8)	49 (23.7)	0.372^**a**^
*B12*			
* High*	3 (17.6)	17 (8.2)	0.378^a^
* Normal*	12 (70.6)	165 (79.7)	
* Low*	2 (11.8)	25 (12.1)	
*Homocysteine*			
* < 10*	7 (41.2)	83 (40.1)	0.201^a^
* 10–15*	5 (29.4)	95 (45.9)	
* > 15*	5 (29.4)	29 (14.0)	
*C4*			
* Low*	5 (29.4)	41 (19.8)	0.326^a^
* Normal*	11 (64.7)	160 (77.3)	
* High*	1 (5.9)	6 (2.9)	

Co-morbidity: Thrombophilia + Diabetes mellitus (DM) + Autoimmune disease + Chronic inf. disease, *p < 0.05 (bold)

a Fisher’s exact test

b Yates Continuity test

Immunological problems, including autoimmune and chronic inflammatory diseases, were significantly more frequent in women with GER than in those without GER (64.7% vs. 11.1%, *p* < 0.001). When evaluated in detail, autoimmune diseases were observed in 35.3% of women with GER compared with 7.2% in the GER-negative group, corresponding to an approximately fivefold higher prevalence in the GER-positive group (*p* = 0.002). Additionally, chronic inflammatory diseases were more common among women with GER than among those without GER (35.3% vs. 5.3%, *p* = 0.001). Although the prevalence of type 2 DM was higher in GER-positive women than in GER-negative women (29.4% vs. 13.5%), this difference did not reach statistical significance (*p* = 0.144). Similarly, no statistically significant difference was observed between women with and without GER in terms of thrombophilia (11.8% vs. 23.7%, *p* = 0.372). In addition, no significant differences were found for biochemical markers, including vitamin B12, homocysteine, or C4 levels (*p* = 0.378, *p* = 0.201, and *p* = 0.326, respectively).

The distribution of *MTHFR* 677 C > T (rs1801133), *MTHFR* 1298 A > C (rs1801131), and PAI-1 4G/5G polymorphisms (rs1799768) was compared between women with and without GER. A statistically significant difference was observed in the distribution of MTHFR 677 C > T genotypes (CC, CT, and TT) according to GER status (*p* = 0.036). The frequency of the *MTHFR* 677TT genotype was higher among women with GER compared with those without GER (0.294 vs. 0.130). However, allelic frequencies did not differ between groups (*p* = 0.792). For the *MTHFR* 1298 A > C and PAI-1 polymorphisms, neither genotype nor allelic distributions differed significantly between the GER-positive and GER-negative groups. Detailed results are presented in Table 4.

**Table 4:** Distribution of *MTHFR* 677 C > T, *MTHFR* 1298 A > C, and *PAI* polymorphisms in women with and without GER

	GER (+) *n* = 17	GER (˗) *n* = 207	*p*
MTHFR 677 C > T			
* CC*	8 (0.471)	73 (0.353)	0.036*
* CT*	4 (0.235)	107 (0.517)	
* TT*	5 (0.294)	27 (0.130)	
*Allelic frequencies*			
* C*	20 (0.588)	253 (0.611)	0.792
* T*	14 (0.412)	161 (0.389)	
*MTHFR 1298 A > C*			
* AA*	7 (0.412)	77 (0.372)	0.938
* AC*	8 (0.470)	104 (0.502)	
* CC*	2 (0.118)	26 (0.126)	
*Allelic frequencies*			
* A*	22 (0.647)	258 (0.623)	0.782
* C*	12 (0.353)	156 (0.377)	
*PAI*			
* 4G4G*	4 (0.235)	27 (0.130)	0.441
* 4G5G*	3 (0.176)	41 (0.198)	
* 5G5G*	10 (0.588)	139 (0.672)	
*Allelic frequencies*			
* 4G*	11 (0.324)	95 (0.229)	0.214
* 5G*	23 (0.676)	319 (0.771)	

Fisher’s exact test, *p < 0.05 (bold), Values are expressed as n (frequency)

## Discussion

The purpose of this study was to evaluate inflammation-related co-morbidities known to be risk factors for obstetric complications in women with GER and a history of poor obstetric outcomes. Thus, the presence of GER may be used to identify cases for the screening of risk factors and to take pre-pregnancy precautions in order to prevent related complications in subsequent pregnancies. In this study, we demonstrated that chronic inflammatory and autoimmune diseases were statistically significantly more frequent in women with GER. In addition, although overall allelic frequencies did not differ between groups, the *MTHFR* 677TT genotype was observed more frequently among women with GER. Taken together, these results suggest that GER in women with poor obstetric history might coexist with systemic inflammatory and genetic/epigenetic factors that are themselves associated with adverse obstetric outcomes.

It has previously been reported that pathological conditions, such as chronic inflammatory diseases, autoimmune disorders, and *MTHFR* polymorphisms, are risk factors for placenta-related obstetric complications [16–31]. In these conditions, cellular structures within the intervillous space of the placenta are likely damaged by toxic substances, including inflammatory cytokines, interleukins, complement system elements, and amino acids (e.g., hyperhomocysteinemia). The resulting placental inflammation might be a contributing factor to obstetric complications and poor gestational outcomes [17–19, 21, 22, 31]. On the other hand, GER is often associated with local inflammatory changes in gastroesophageal tissues (e.g., esophagitis) and may also be accompanied by systemic inflammatory responses, potentially reflected by alterations in circulating inflammatory mediators [4–6]. Both GER and immune system problems (autoimmune diseases and chronic inflammatory diseases) seem to be connected to each other, with each disorder augmenting the other through systemic inflammation.

Given the potential role of systemic inflammation as a link between GER and immune dysregulation, we also investigated genetic factors that could influence inflammatory and thrombotic pathways. In this study, a higher frequency of the *MTHFR* 677TT genotype was observed in women with GER, whereas no significant association was observed for the 1298 A > C polymorphism. The observed differences between *MTHFR* C677T and A1298C polymorphisms could reflect their distinct biological effects. The C677T variant results in a thermolabile enzyme with a greater reduction in *MTHFR* activity and a more pronounced disruption of folate-homocysteine metabolism, whereas the A1298C variant is generally associated with a milder reduction in enzyme function [34].

Chronic inflammatory changes in the esophagus have been associated with epigenetic alterations in mismatch repair genes, such as human mutL homolog 1 (hMLH1) [11]. Promoter hypermethylation has been proposed as a key regulatory mechanism of hMLH1 expression, and previous studies have reported increased hMLH1 hypermethylation in patients with GER, suggesting that the reflux-induced local inflammatory microenvironment may promote such epigenetic modifications [11]. DNA methylation is closely linked to the folate-homocysteine metabolic pathway, in which *MTHFR* plays a central role [35, 36]. In this study, the *MTHFR* 677TT genotype was observed more frequently in women with GER; however, no significant differences were detected in circulating vitamin B12, homocysteine, or C4 levels between groups. This finding suggests that the observed genetic association may not be directly reflected by systemic biochemical markers in this cohort. It is therefore plausible that the *MTHFR* 677TT genotype might be involved through local, tissue-specific mechanisms rather than systemic biochemical alterations. Previous studies have also reported altered DNA methylation patterns in genomic regions involved in inflammatory pathways that contribute to both innate and adaptive immune responses [37]. In this context, it may be hypothesized that systemic inflammation represents a shared biological background linking GER with placental inflammatory processes. Nevertheless, the precise mechanisms underlying these associations remain to be elucidated and warrant further investigation.

The main limitations of this study are its retrospective design and the limited sample size, particularly in the GER-positive group, which reduce statistical power, limit comprehensive multivariate adjustment, and causal interpretation. Furthermore, the lack of direct measurements of inflammatory and immune markers limits mechanistic interpretation. Despite these constraints, this is the first study to investigate the association between GER and poor obstetric history. Future prospective studies with larger, multicenter, and more balanced cohorts and detailed biomarker assessments are needed to confirm our findings and elucidate the underlying mechanisms.

## Conclusion

In conclusion, chronic inflammatory disease and autoimmune disorders were significantly more frequent in women with GER and poor obstetric history. Furthermore, a higher frequency of the *MTHFR* 677TT genotype was observed in women with GER. The presence of GER may be associated with inflammation-related, placenta-associated obstetric complications and adverse gestational outcomes in subsequent pregnancies. Therefore, pre-conceptional counseling may be beneficial for women with GER and a poor obstetric history.

## Data Availability

The data of this study are available from the corresponding author upon reasonable request.
